# Plastic surgery involvement in diabetic foot care: A national survey in National Health Service England

**DOI:** 10.1016/j.jpra.2025.10.025

**Published:** 2025-10-25

**Authors:** Rachel H Enright, Isabella Stevens-Harris, James KK Chan

**Affiliations:** a Plastic Surgery Department, St Vincent’s Hospital, Dublin, Ireland; b Royal College of Surgeons, Dublin, Ireland; c Department of Plastic Surgery, Stoke Mandeville Hospital, Buckinghamshire Healthcare NHS Trust, Aylesbury, England, United Kingdom; d Nuffield Department of Orthopaedics, Rheumatology and Musculoskeletal Sciences, Oxford, England, United Kingdom

**Keywords:** Diabetes, Lower limb, Limb salvage, Reconstructive surgery

## Abstract

Diabetes is the leading cause of major lower limb amputation in the United Kingdom.

The cost of Diabetic Foot Disease in England is estimated to exceed £1 billion annually, driven largely by the treatment of foot ulcers and lower limb amputations. Flap reconstruction has been shown to improve limb salvage. The role of Plastic Surgery remains poorly defined in the major leading guidelines on diabetic foot care.

A cross-sectional survey was distributed via email to eligible plastic surgery units in NHS England to ascertain the current landscape of involvement in diabetic foot care.

Responses were received from 79 % eligible units. Among these, 70 % reported no routine Plastic Surgery representation in their Multidisciplinary Diabetic Foot Team (MDFT). However, 84 % reported receiving at least one referral per annum for diabetic foot-related issues, and 40 % units reported providing 11 or more ad hoc consultations per year. Referrals originated predominantly from orthopedics and vascular surgery. The most frequent reason for referral (65 %) was soft tissue reconstruction following foot ulceration. Notably, 59 % of units reported encountering cases involving failed soft tissue reconstructions initially performed by non-plastic surgeons at least once a year.

Plastic Surgery plays a vital yet under-recognized role in diabetic foot management. This survey highlights the need for formalized plastic surgery specialist input in complex soft tissue reconstruction within diabetic foot care.

## Introduction

Diabetic foot disease (DFD) represents one of the most serious and costly complications of diabetes, with significant implications for both patient outcomes and healthcare systems. In the UK, over 200,000 people are diagnosed with diabetes each year, alongside a concerning rise in type 2 diabetes among children and adolescents.^1^ For individuals with diabetes, the lifetime risk of developing a foot ulcer is estimated to be as high as 34 %.^2^ These ulcers frequently progress to infection, hospitalization, and ultimately amputation—outcomes that are not only devastating for patients but also place a substantial financial burden on the healthcare economy.

The cost of DFD to National Health Service (NHS) England is estimated to exceed £1 billion annually, driven largely by the treatment of foot ulcers and lower limb amputations.^3^ Diabetes is now the leading cause of major lower limb amputation in the UK,^4^^,^^5^ yet up to 80 % of these procedures are considered preventable through timely intervention and coordinated care.^6^ Early access to multidisciplinary services plays a critical role in improving clinical outcomes and reducing these avoidable complications.^7^ Moreover, limb preservation is associated with improved quality of life, increased life expectancy, and reduced healthcare costs, particularly in patients with co-existing peripheral arterial disease.^8^^,^^9^

In 2019, the Department of Health and Social Care launched a national initiative to reduce amputation rates through early intervention and coordinated care. Central to this initiative is the implementation of Multidisciplinary Foot Services (MDFS), which integrates the expertise of endocrinologists, podiatrists, vascular surgeons, orthopedic surgeons, and other specialists. Recent national guidance—including the joint position statement by the British Orthopaedic Association (BOA), British Orthopaedic Foot & Ankle Society (BOFAS), Vascular Society, and Diabetes UK—reinforces the importance of including clearly defined surgical roles within these teams. However, plastic surgery is notably absent from these recommendations.^10^ Their role remains undefined in both NICE guidelines and international frameworks, including the International Working Group on the Diabetic Foot guidelines.^11^

To address this gap, the British Association of Plastic, Reconstructive and Aesthetic Surgeons (BAPRAS) established a Special Interest and Advisory Group (SIAG) in 2023. This study, undertaken on behalf of the SIAG, aims to map current practice, assess barriers to integration, and inform future service development to improve patient outcomes and optimize resource use.

## Methodology

A mixed-method analysis was performed, combining quantitative descriptive statistics and qualitative thematic analysis. A national cross-sectional survey was conducted to assess the extent and nature of plastic surgery involvement in diabetic foot care across England.

A structured online survey questionnaire was developed using Google Forms by the Diabetic Foot Special Interest and Advisory Group of the British Association of Plastic, Reconstructive and Aesthetic Surgeons (BAPRAS). Following internal piloting, it was distributed via email to all NHS plastic surgery units in England identified as being part of an established diabetic foot Multidisciplinary Diabetic Foot Team (MDFT), *n* = 47. Units serving exclusively pediatric or cancer populations were excluded.

For the purposes of this study, the “diabetic foot” was defined as a pathology involving diabetic foot ulcers or acute diabetic foot infections (also known as “foot attacks”). Traumatic injuries, elective foot surgery or complications unrelated to diabetic foot disease were excluded.

**Figure 1:**
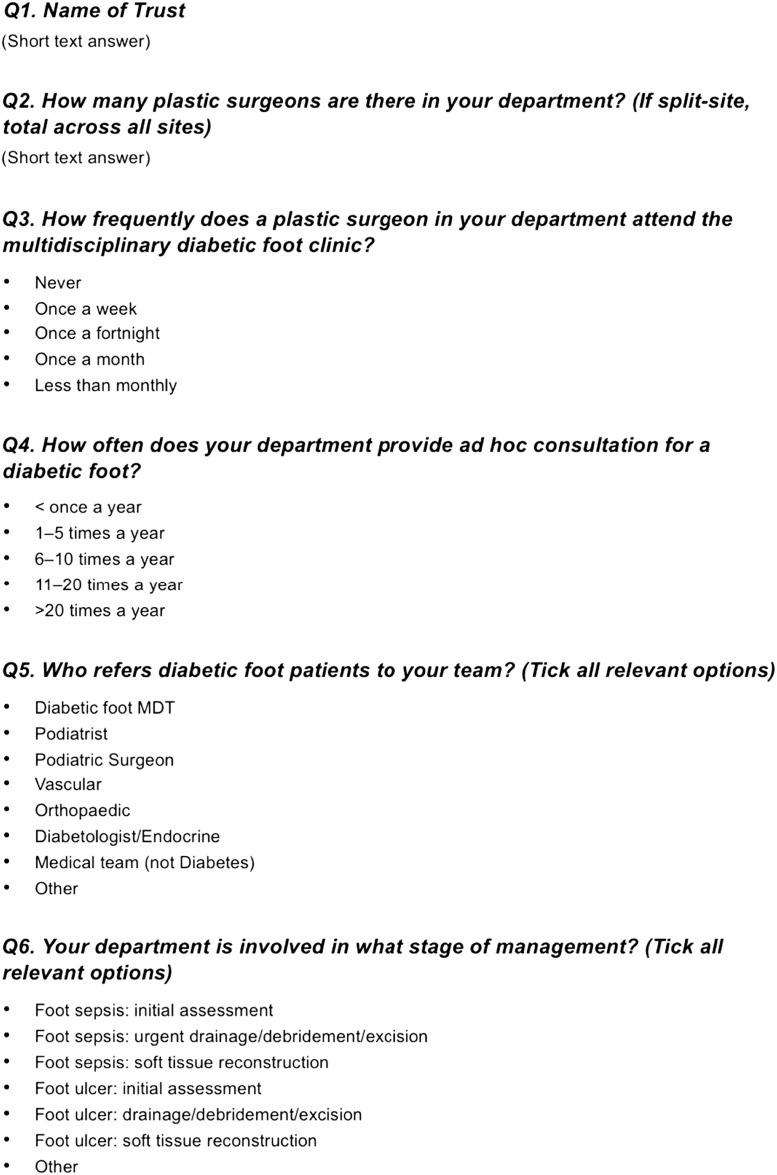

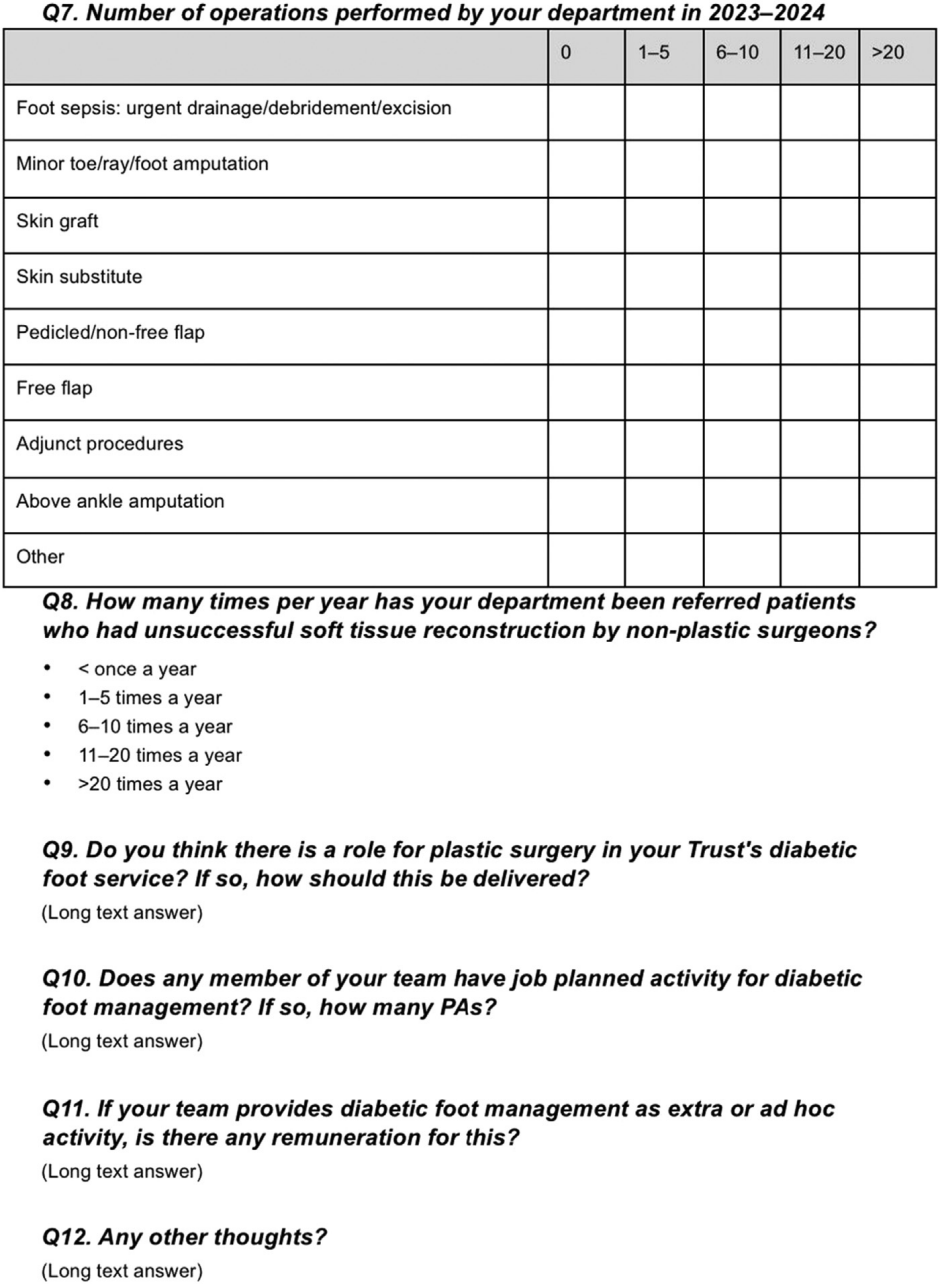
1a and 1b. The survey. A copy of the questions on the online survey which was sent to participants.

A copy of the survey is shown in Figures 1a and 1b We permitted one response per unit and sought to include, where possible, the response from the lower limb reconstructive surgeon, and where not possible, the clinical lead (i.e. head of department).

The questionnaire collected data on: (i) the presence of plastic surgeons in diabetic foot MDTs, (ii) referral patterns, (iii) the volume of annual consultations to plastic surgery services for diabetic foot pathology, (iv) access to dedicated theatre capacity, and (v) perceived barriers and enablers to plastic surgery involvement. Respondents were also asked to identify referral triggers and whether reconstructive services were delivered through formalized pathways or on an ad hoc basis.

Quantitative data from multiple-choice, numeric, and checkbox responses were analyzed descriptively using frequencies and percentages and stratification. Free text responses were subjected to qualitative thematic analysis, with themes extracted using ChatGPT-5 to identify recurrent patterns.

## Results

### Response rate, MDT involvement and demand for plastic surgery services

**Figure 2:**
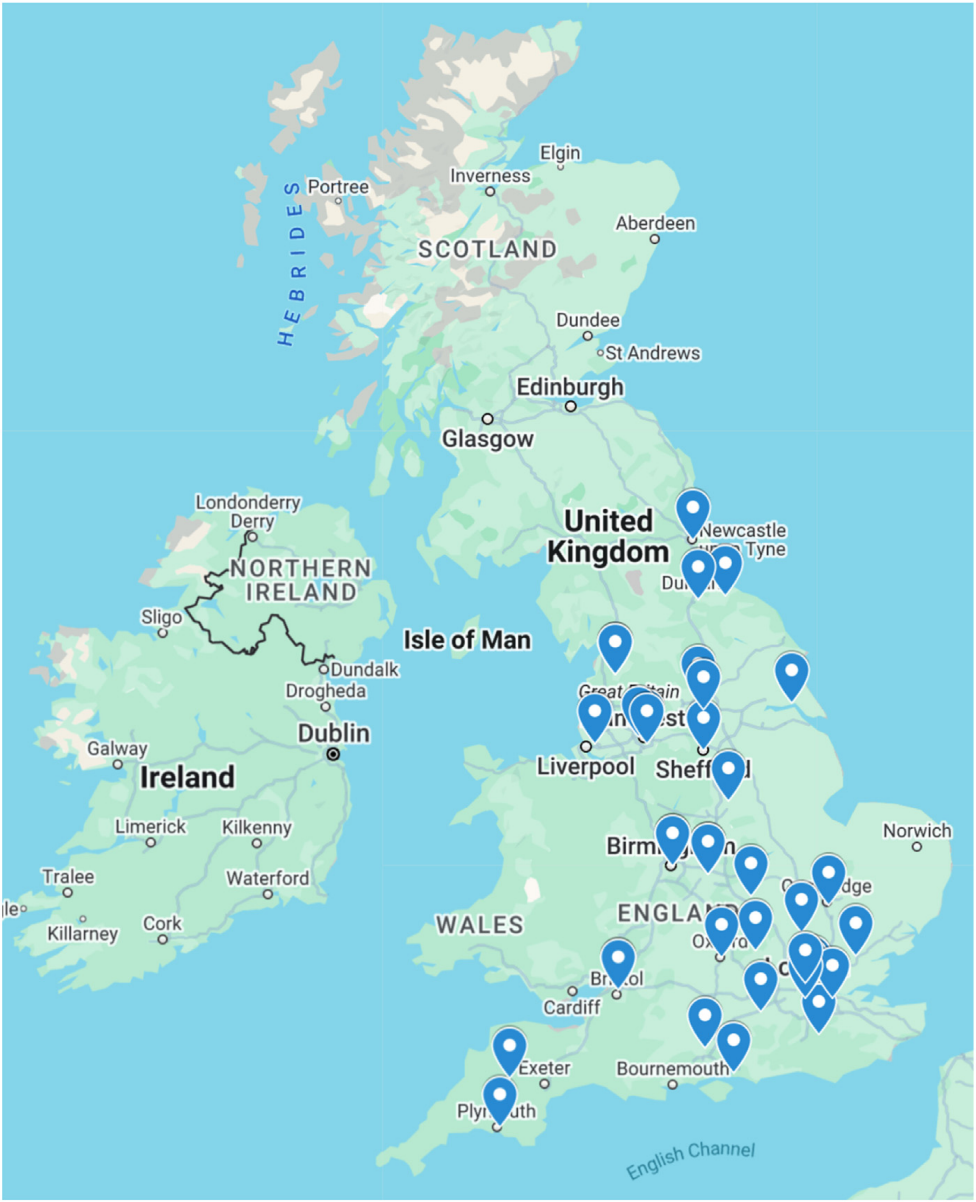
A map of the United Kingdom highlighting the plastic surgery units in England that responded to our survey. See Table 1 for further detail.

Of the 47 eligible NHS plastic surgery units across England, 37 completed the survey, yielding a 79 % response rate. Each unit will be part of an NHS trust. An NHS trust is an organisational unit within the NHS that provides healthcare services including running hospitals to a specific geographic region. The geographical distribution of responding units is illustrated in Figure 2 and Table 1.

**Table 1:** List of units which responded.

Addenbrooke’s Hospital, Cambridge	Northern Care Alliance
Barts Health NHS Trust	Northumbria Healthcare NHS Trust
Buckinghamshire NHS Trust	Nottingham University Hospitals NHS Trust
Chelsea and Westminster NHS Foundation Trust	North West Anglia Foundation Trust Hospitals
County Durham and Darlington NHS Trust	Oxford University NHS Trust
East and North Hertfordshire NHS Trust	Portsmouth Hospitals University NHS Trust
Frimley Health NHS Foundation Trust	Queen Victoria Hospital
Guy's & St Thomas' NHS Foundation Trust	Royal Devon University Healthcare Trust
Hull University Teaching Hospitals NHS Trust	Royal Free Hospital, London
Imperial College Healthcare NHS Trust	Salisbury Foundation Trust
Lancashire Teaching Hospitals	Sheffield teaching hospitals
Leeds Teaching Hospitals NHS Trust	South Tees Hospitals NHS Trust
Manchester University Foundation Trust	St George's University Hospitals NHS Foundation Trust
Mersey and West Lancashire NHS Trust	Sandwell and West Birmingham Hospitals NHS Trust
Mid and South Essex NHS Foundation Trust	The Christie NHS Foundation Trust
MidYorkshire NHS Teaching Trust	University Hospitals Coventry & Warwickshire
Newcastle Hospitals NHS Foundation Trust	University Hospitals Birmingham NHS Trust
North Bristol NHS Trust	University Hospitals Plymouth NHS
Northampton General Hospital	

**Figure 3:**
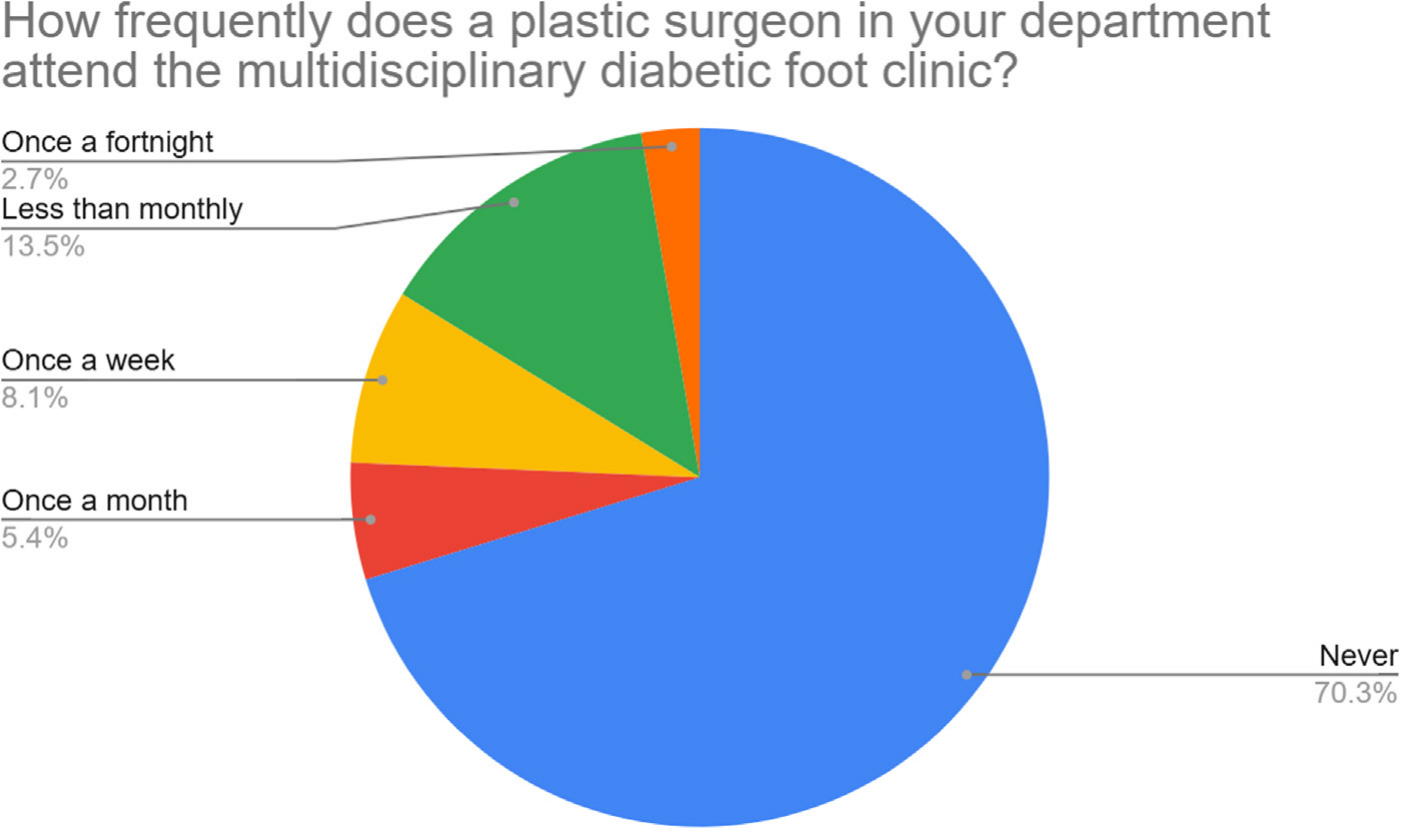
Frequency of plastic surgery attendance at multidisciplinary diabetic foot clinics (*n* = 37). This pie chart demonstrates the frequency of attendance at multidisciplinary diabetic foot clinics according to our survey respondents.

**Figure 4:**
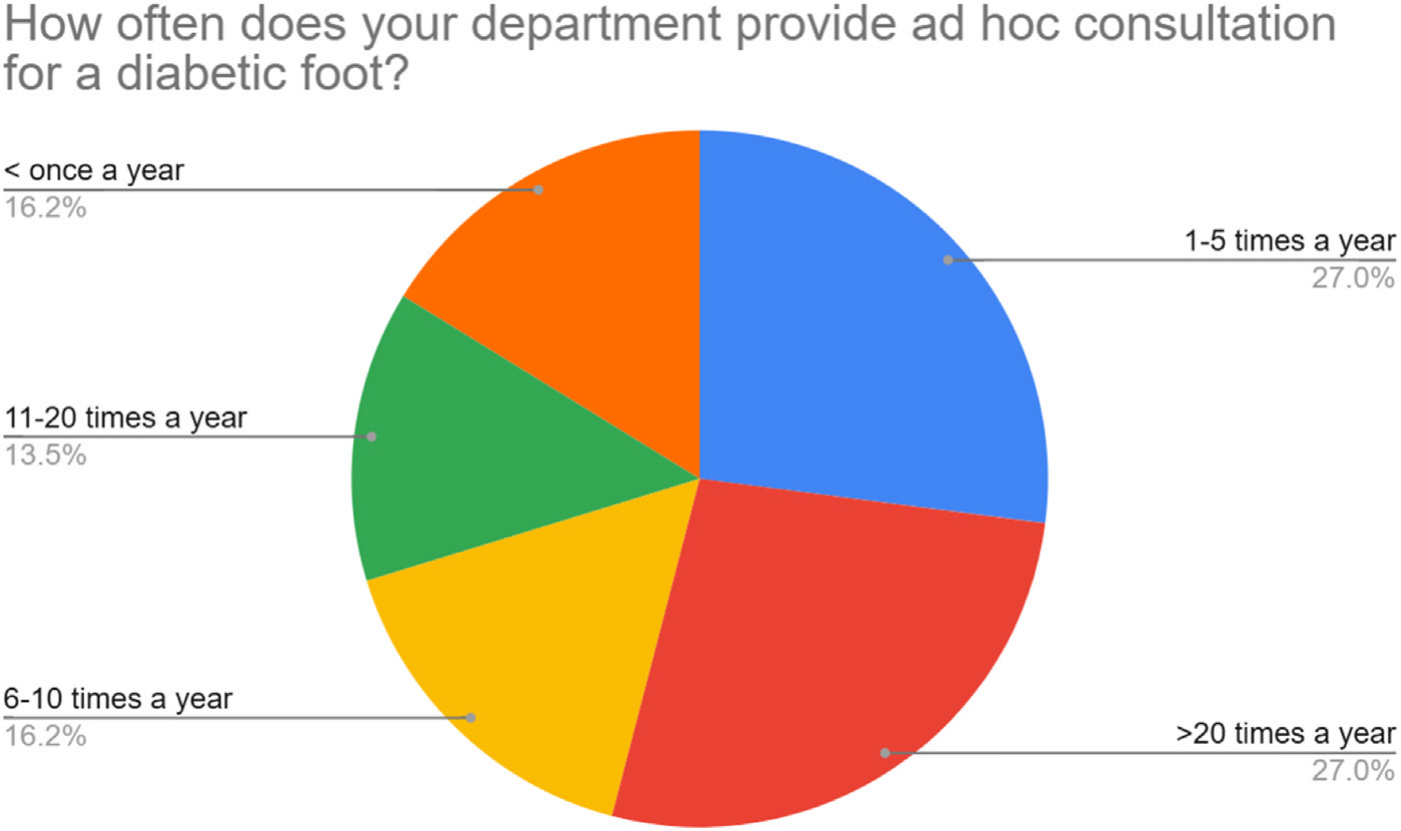
How often does your department provide ad hoc consultation for a diabetic foot? (*n* = 37). This pie chart illustrates how often survey respondents were asked to see a patient outside the setting of a multidisciplinary team meeting or clinic.

**Figure 5:**
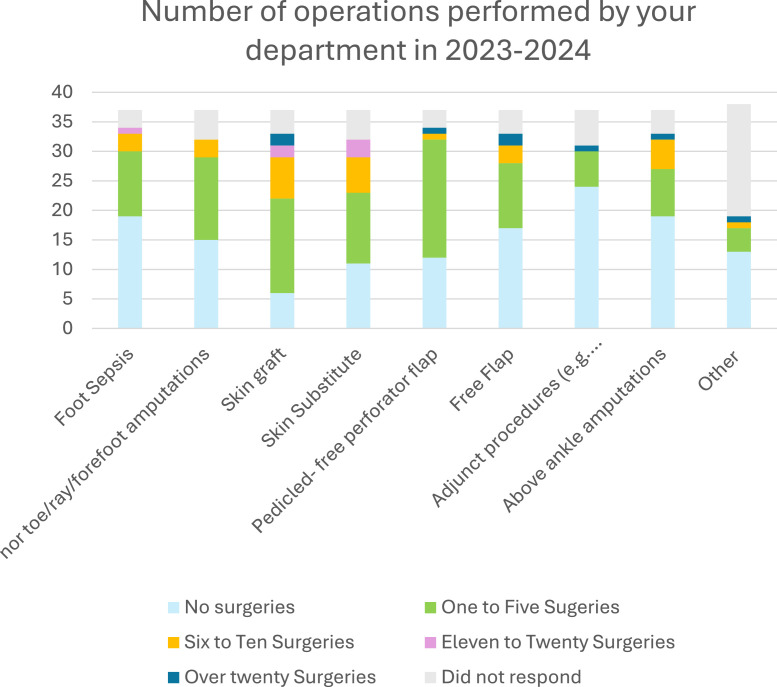
Frequency of surgeries categorized by type of surgery performed. Participants could select from a range of frequencies, each represented by a distinct color (see legend), across various types of procedures displayed on the x-axis.

Among the responding units, 70 % reported that no plastic surgeons routinely attended their local multidisciplinary diabetic foot team (MDFT). Figure 3 shows the frequency of plastic surgery representation at MDFT clinics. Despite this, 84 % of units received referrals related to the diabetic foot at least once a year; >40 % units reported providing 11 or more ad hoc consultations per year, with 27 % of units receiving >20 (Figure 4). Plastic surgeons were involved across all aspects of the diabetic foot journey, including treatment of acute foot sepsis to amputations and soft tissue reconstruction. Within one year, the majority of units did 10 or less operations of each surgery specified.. Figure 5 depicts the frequency of surgical procedures performed, stratified by type of surgery.

### Referral sources and indications

**Figure 6:**
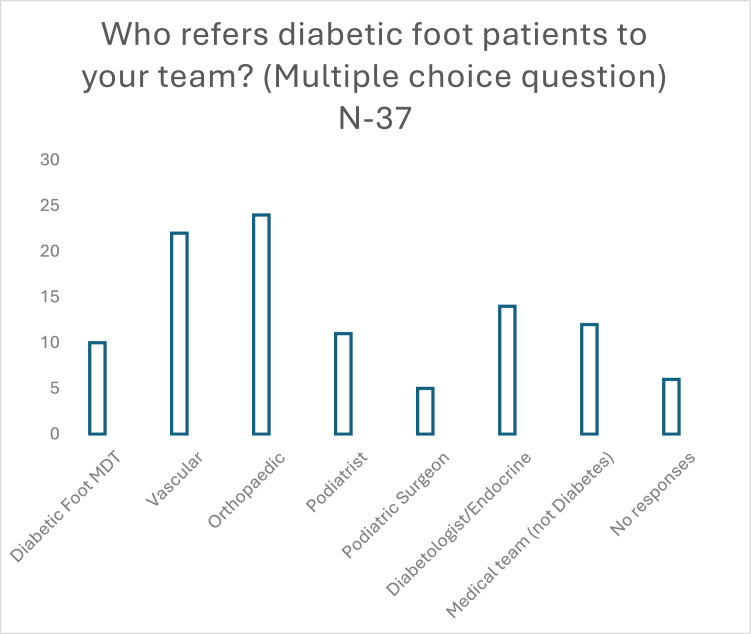
Who refers diabetic foot patients to your team?” (Multiple responses allowed). Respondents could select any speciality from which they received a referral. If the decision to refer was made at MDT then diabetic foot MDT was selected.

When asked the sources of referrals (multiple responses allowed), 65 % of respondents identified orthopedics, 59 % vascular and 27 % MDFT. 16 % received no referrals (Figure 6).

**Figure 7:**
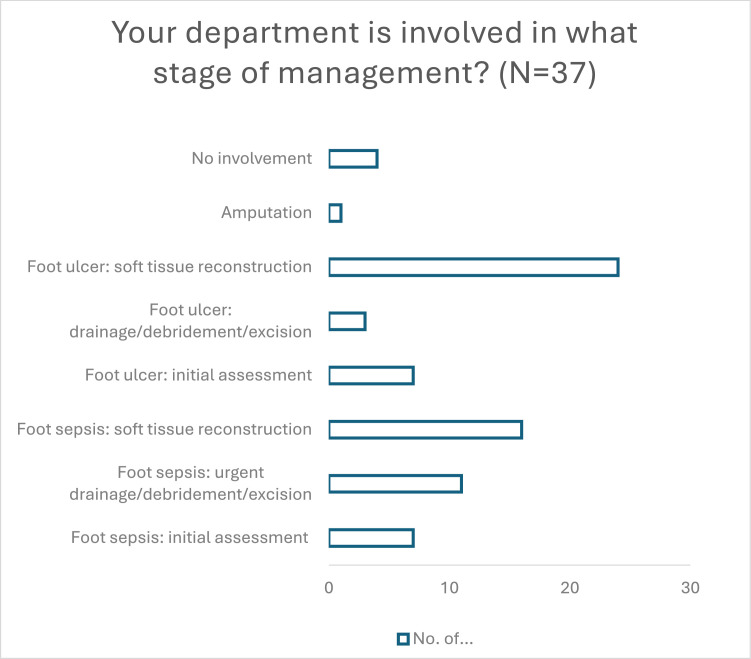
Timing of referrals (multiple responses allowed). Respondents were asked to select from an array of stages of treatment. Respondents could select multiple responses as they may have received more than one type of referral.

The reason for referral was most commonly the consideration of soft tissue reconstruction of diabetic foot ulcers (65 %) and soft tissue reconstruction of foot sepsis (43.2 %). (Figure 7) 19 % of units have also been referred patients requiring an initial assessment of ulcers or foot sepsis, and 29.7 % received a consult to urgently perform drainage/debridement/excision in foot sepsis. About 59 % received at least one annual referral to revise failed soft tissue reconstructions initially performed by non-plastic surgeons.

### Current job allocations

**Figure 8:**
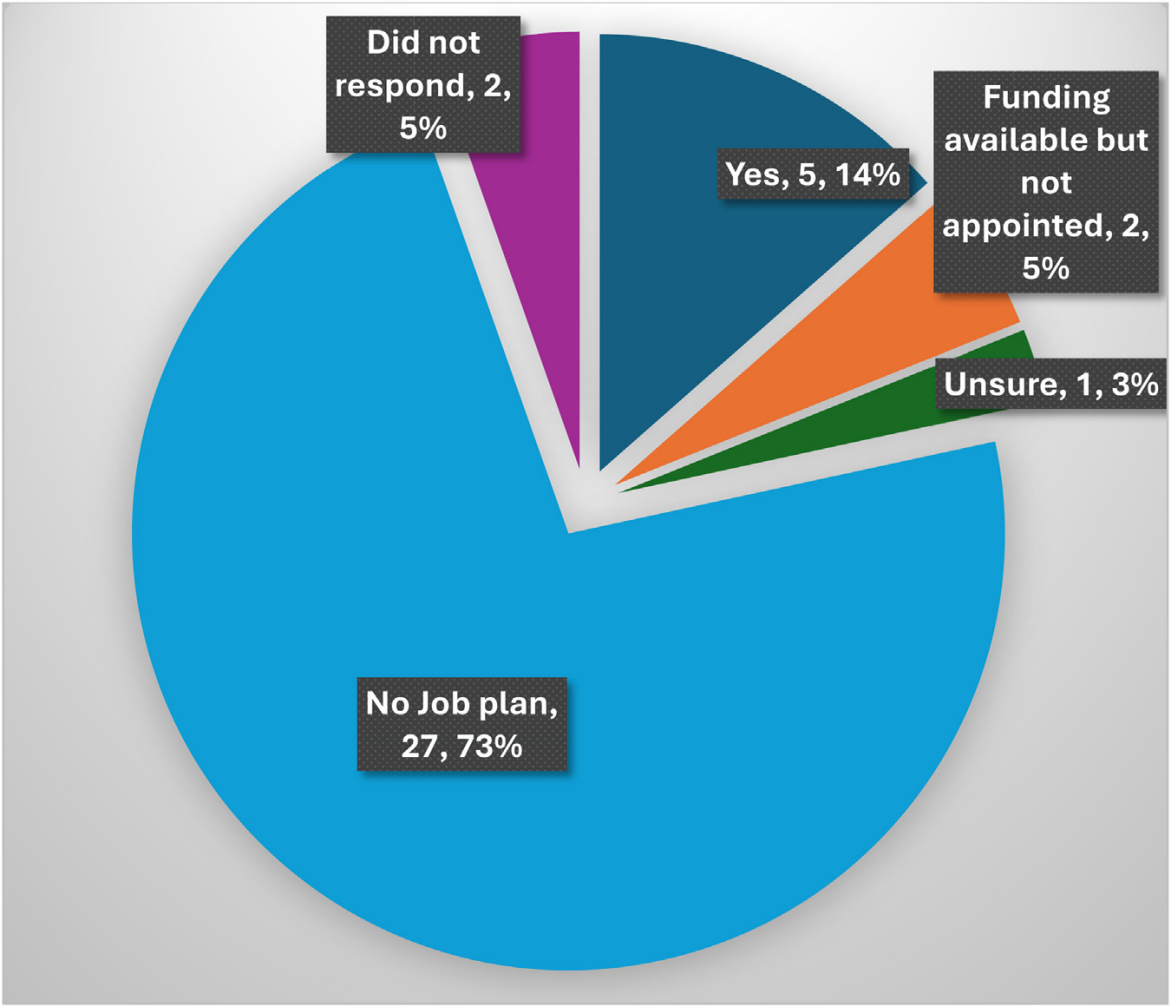
Funding for positions. This pie chart shows the number and percentage of respondents for each answer to the question of whether any member of their team has a job-planned activity related to diabetic foot management.

The majority of units, 73 %, did not have any member of the team with a job planned activity for diabetic foot management; only 14 % did (Figure 8). Of the respondents that did have programmed activity plans specifically for diabetic foot reconstruction, one unit had 0.25 PA, another unit had one session per week for two consultants in parallel with other commitments, and another had one per month. (In the NHS, a programmed activity (PA) is a standard 4-h block of work that forms the basis of a doctor’s contract and job plan.)

## Thematic analysis

**Figure 9:**
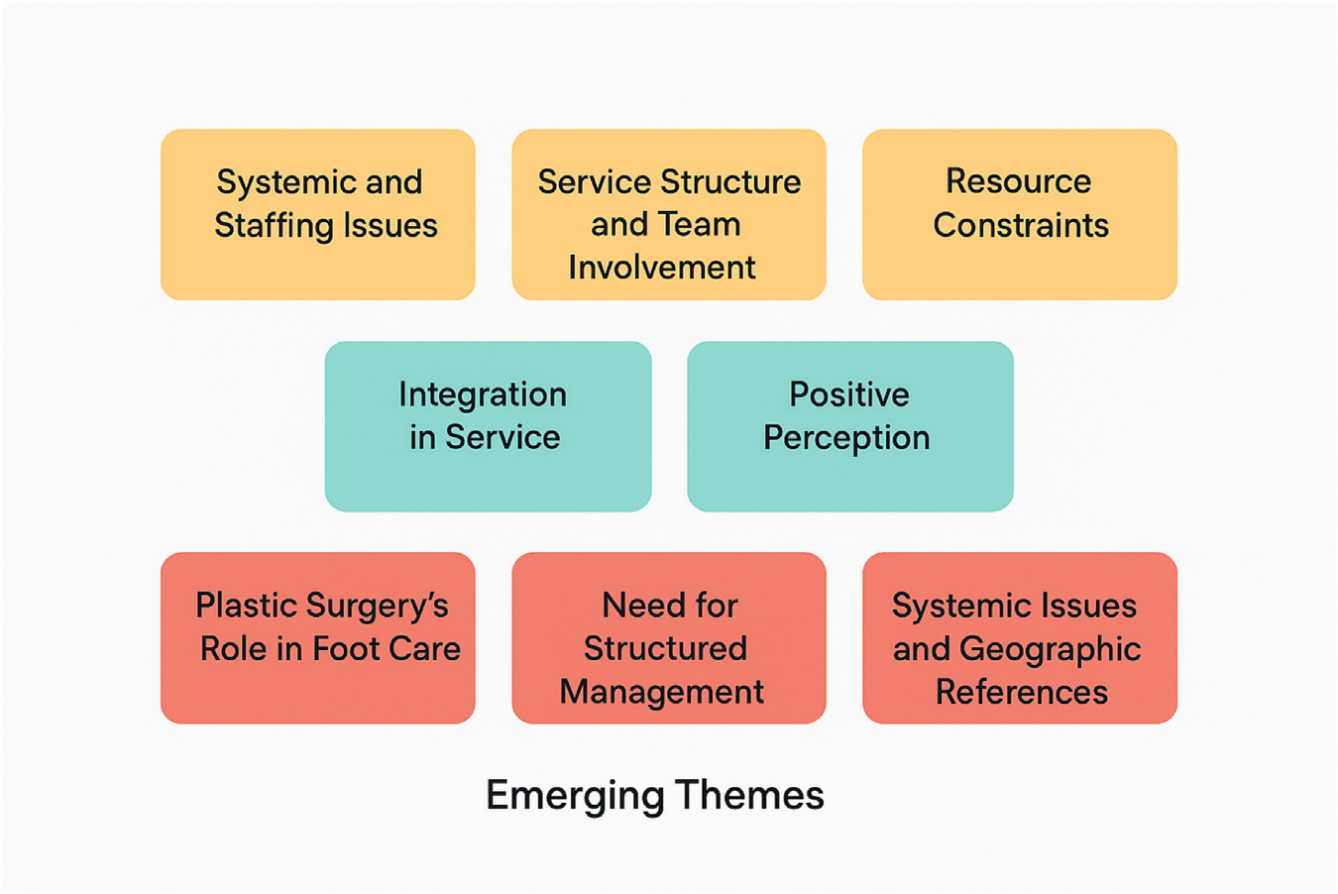
Thematic analysis of open ended questions. This diagram illustrates the most common themes which emerged from three questions which respondents were asked to fill in a free-text section. The responses in Yellow correspond to the question “*What are the current barriers in your Trust to offering more plastic surgical input for people with diabetic feet?”.* The responses in Blue correspond to “*Do you think there is a role for plastic surgery in your Trust's diabetic foot service? If so, how should this be delivered?”.* The responses in Red correspond to “*Any other thoughts?”.*

The main themes identified are shown in Figure 9.

### What are the current barriers in your trust to offering more plastic surgical input for people with diabetic feet?

Respondents highlighted issues such as inadequate referral pathways, limited engagement from multidisciplinary teams, insufficient staffing, and resource limitations (e.g., operating theatre time, funding).

### Do you think there is a role for plastic surgery in your trust's diabetic foot service? If so, how should this be delivered?

Most respondents support the integration of plastic surgery in diabetic foot care, suggesting early involvement in MDTs and regular clinic participation. Some responses reflect Trust-specific perspectives or current service limitations.

### Any other thoughts?

Additional comments emphasize the importance of structured service delivery, patient education, and national disparities. Some also reflect ongoing systemic or regional service gaps.

## Discussion

Plastic surgery remains an under-recognized yet potentially critical component in the multidisciplinary management of diabetic foot disease.

In a recent survey of orthopedic foot and ankle surgeons, 111 of 117 Trusts (95 %) had a Multidisciplinary Diabetic Foot Team (MDFT) .^4^ Podiatrists were present in all MDFTs, a diabetologist in 95 %, a vascular surgeon in 58 % and an orthopedic surgeon in 45 %. In another survey of 46 units that provided an MDFT, vascular surgeons attended 63 % of the sessions, and orthopedic surgeons attended 26 % of the sessions in the 23 units that met once weekly.^12^ Neither survey reported the presence of plastic surgeons.

Our survey found that plastic surgery units in NHS England are not routinely involved in the care of people with diabetic foot problems. When referrals are made to plastic surgery, these tend to be on an ad hoc basis. Plastic surgical input is also predominantly sought for reconstructive rather than ablative procedures. They are often asked to manage previously failed reconstructive attempts.

With the specific expertise in soft tissue reconstruction and complex wound management, routine participation by plastic surgeons can potentially contribute to better outcomes and a reduction in the number of amputations. There has been a growing interest within the plastic surgical community in diabetic foot reconstruction. Reported limb salvage rates following free flap reconstruction for diabetic lower limb defects is 90 % for diabetic foot ulcers although this may be lower in the acutely septic foot.^13^, ^14^, ^15^

An important observation is that plastic surgery is rarely asked to participate in the initial assessment. This may indicate a lost opportunity to offer a joint opinion and plan for future salvage reconstruction. By contrast, a joint orthoplastic assessment is the current gold standard in the management of open or complex fractures.^16^^,^^17^ In patients with non-healing ulcers or deep tissue infections, early involvement of reconstructive services may prevent progression to major amputation. An orthoplastic approach has recently been shown to improve outcomes of healing, major amputation rate and recurrence rates.^18^

Our survey also highlights persistent structural and educational barriers to the integration of plastic surgery in diabetic foot care pathways. Respondents identified the lack of recognition, inadequate referral pathways, and logistical challenges as major obstacles. Similar to the initial exclusion of orthopedic surgery from national DFD guidelines and audits,^4^ plastic surgery remains underrepresented, impeding service development and workforce planning. The lack of education is also a significant barrier. For example, diabetic foot reconstruction is not featured as a core part of the plastic surgery higher surgical training curriculum in the UK.^19^

Consideration to include diabetic foot reconstruction as a defined subspecialty area to reflect the modern reconstructive challenges of the society. There is also a lack of awareness of the role of plastic surgery amongst other specialists on the MDFT. Fortunately, there is now a growing awareness of this expanding subspecialty with sessions dedicated to plastic surgical reconstruction in national and international diabetic foot meetings. Development of evidence-based guidelines, supported by commissioning levers, is crucial to incentivize and embed plastic surgery involvement at the Trust and system level. Hence, future research should focus on quantifying the demand of diabetic foot cases that would benefit from plastic surgical intervention and evaluating the cost-effectiveness of earlier reconstructive input to prevent amputations or the management of chronic wounds. This would enable the establishment of service targets and inform appropriate funding, training, and consultant job planning. Including and establishing a clearly defined role for plastic surgeons in MDFT will help to standardize care, improve outcomes, guide workforce planning and enhance the efficiency of NHS resource utilization.

This study has several limitations. First, the authors’ professional interest in this area may introduce confirmation bias. While every effort was made to report findings objectively, this potential influence should be considered. Second, the survey design relied on self-reported data from designated respondents at each unit, thus introducing reporting bias and does not allow independent verification of service activity. Third, the response rate was 77 % and the survey included only centers in NHS England. Non-response meant incomplete national coverage and potential selection bias. Fourth, restricting the survey to one response per unit may have limited the capture of different perspectives within teams despite seeking to identify the best placed respondent on each team. Finally, the cross-sectional design provides a snapshot in time and may not reflect evolving service provision or practice changes.

## Conclusions

As the burden and complexity of diabetic foot pathology increase, the case for routine involvement of plastic surgery within MDFT is increasingly compelling. However, the current landscape of NHS England is one where the demand for plastic reconstructive surgical intervention is either unmet or mostly provided sporadically on an ad hoc basis. Whilst the majority of responders supported the integration of plastic surgery within MDFTs, a number of barriers, including the lack of referral pathways and resource constraints, have been highlighted.

## Ethical approval

Not applicable.

## Funding

The authors received no funding for carrying out this research.

## Declaration of generative AI and AI-assisted technologies in the writing process

The authors used ChatGPT-5 in order to assist with the generation of thematic analysis. After using this tool/service, the authors reviewed and edited the content as needed and take full responsibility for the content of the publication.

## Declaration of competing interest

We have no conflicts of interest. James Chan is a deputy editor of the JPRAS and was not involved in the editorial review or the decision to publish this article.
